# Evaluation of malnutrition risk screening in hospitalized children with digestive system diseases—a single-center cross-sectional study

**DOI:** 10.3389/fped.2025.1598962

**Published:** 2025-07-17

**Authors:** Xiaoyu Li, Yu Zou, Jing Zuo, Tian Xu, Huilin Zeng, Ruiju Ou, Dongling Dai

**Affiliations:** Department of Gastroenterology, Shenzhen Children’s Hospital, Shenzhen, Guangdong, China

**Keywords:** nutrition risk screening, nutritional intervention, modified pediatric malnutrition risk screening tool, hospitalized children, screening tool for the assessment of malnutrition in pediatrics (STAMP)

## Abstract

**Objective:**

This study aimed to assess the malnutrition risk of hospitalized children with digestive system diseases and provide evidence for clinical nutritional support.

**Methods:**

In this single-enter cross-sectional study, the modified pediatric malnutrition risk screening tool was used to assess the malnutrition risk of pediatric patients hospitalized for digestive system diseases from January 2024 to June 2024. The screening was carried out within 24 h after admission, and scores ≥4 and <4 were considered as high risk of malnutrition and non-high risk of malnutrition, respectively. We collected the data from all children, including age, gender, malnutrition risk, nutritional support, and clinical outcomes. SPSS software package (version 23.0) was used for data processing.

**Results:**

A total of 1,200 children aged 1 month to 18 years were included in this study. The incidence of malnutrition risk in all hospitalized children was 53.17%, and the percentage of high malnutrition risk was 13.67%. There was a significant difference in the incidence of high malnutrition risk between different diseases (*P* < 0.01). The difference in nutritional support rate (*P* < 0.05) and outcomes (*P* < 0.05) was also significant in patients with different degrees of malnutrition risk.

**Conclusions:**

Timely, standardized, and comprehensive nutritional assessment is of crucial importance for reasonable nutritional interventions to improve clinical outcomes in children with high malnutrition risk.

## Introduction

Malnutrition is prevalent in hospitalized children. It may affect the growth and development of children, aggravate the infection rate and mortality of hospitalized children, prolong hospitalization time, and increase medical expenses (1). Previous studies showed that one-third of the annual 6.9 million deaths in children under the age of 5 were due to malnutrition (2, 3). The incidence of malnutrition in developing countries is higher (4). The incidences of malnutrition and nutritional risk in hospitalized children in China were approximately 15%–50% and 57.28%–81.26%, respectively (5, 6), while the incidence of malnutrition in hospitalized children in Thailand was as high as 50%–60% in 1995 (7). A study screening nutritional risk for 2,567 hospitalized children from 14 hospitals in 12 European countries found that the incidence of nutritional risk was 23% (8). Lama et al. (9) found that 48.4% of 250 hospitalized children were at risk of malnutrition in screening. Nutritional risk screening is the first step in clinical nutrition management. Through a quick and easy nutritional risk screening tool, children at risk of malnutrition can be identified early, and targeted and reasonable nutritional support will reduce complications, shorten the length of hospital stay, and improve clinical prognosis.

There are five nutritional screening tools commonly used for hospitalized children, namely, Pediatric Nutrition Risk Score (PNRS) (10), Screening Tool for the Assessment of Malnutrition in Pediatrics (STAMP) (11), Pediatric Nutrition Screening Tool (PNST) (12), Screening Tool for Risk on Nutritional Status and Growth (STRONGkids) (13), and Pediatric Yorkhill Malnutrition Score (PYMS) (14). Chinese experts from the pediatric group, the Society of Parenter and Enteral Nutrition, Chinese Medical Association, established a screening tool for Chinese hospitalized children based on STRONGkids and STAMP, named the modified pediatric malnutrition risk screening tool (MPMRST) (15). This screening tool integrates the characteristics of the pediatric disease spectrum and the requirements of nutritional screening in China, with a sensitivity of 82% (95% CI: 76%–87%) and a specificity of 71% (95%CI:67%–74%). It is easy to operate, and clinical personnel can complete the assessment of the nutritional risk of children within 3 min. Li Xinyi (5) and Jinye et al. (15) once used this tool in their research to conduct nutritional risk screening on 16,249 and 2,632 hospitalized children, respectively. It can effectively screen out the risk of malnutrition in hospitalized children and demonstrate good clinical predictability. In recent years, this modified screening tool for the assessment of malnutrition in children has been reported to be effective in identifying the risk of malnutrition in hospitalized children and in better predicting clinical outcomes. Previous studies have focused on all hospitalized children; however, there are rarely studies on its application in the pediatric population with digestive diseases (4, 5, 16). In this study, we investigated the results of malnutrition risk screening and clinical outcomes of children hospitalized in the gastroenterology department of Shenzhen Children's Hospital, China, by using a modified version of the screening tool. We aimed to evaluate its early identification of high malnutrition risk and provide evidence for nutritional intervention.

## Subjects and methods

### Subjects

All children hospitalized in the gastroenterology department of Shenzhen Children's Hospital from January 2024 to June 2024 were included in this study, and parents or guardians consented to participate in this study. The inclusion criteria included hospitalization time longer than 24 h, age <18 years, and written consent obtained. The exclusion criteria included comorbidity with chronic wasting diseases, critical condition and unstable vital signs, and inability to obtain weight due to pleural effusion, ascites, edema, etc.

### Methods

In this single-center cross-sectional study, the modified pediatric malnutrition risk screening tool was used to assess the malnutrition risk of pediatric patients hospitalized for digestive system diseases from January 2024 to June 2024. The screening was carried out within 24 h after admission. We collected the data from all children, including age, gender, malnutrition risk, nutritional support, and clinical outcomes.

### Quality control

We had a team of malnutrition risk screening consisting of one nutritionist, one gastroenterologist, one head nurse, and two specialist nurses. The final malnutrition risk screening score was determined through three-level quality control to guarantee the accuracy of scores in this study. Within 24 h of admission, an initial malnutrition assessment was performed by a charge nurse, reviewed by a team leader, and finally reviewed by a specialist nurse. Each child’s weight was measured upon admission, with infants and young children weighed after removing clothing and diapers, with an accuracy of 0.1 kg. Children aged 2 years and older were measured by a height–weight scale (RGZ-120 body scale, Shanghai Dongfang Weighing Apparatus Co., Ltd), and children under 2 years old were measured by a length–weight scale (seca 376 baby scale, Seca GmbH, Germany). The height was measured for each child with shoe removal, with an accuracy of 0.1 cm.

### Sample size calculation

This study is a cross-sectional study. According to previous literature reports, the incidence of malnutrition risk is 50%, with a margin of error of 3% and a confidence level of 95%. The minimum sample size calculated is 1,067 cases. Considering the need for subgroup analysis, a total of 1,200 cases were finally included.

### Statistical analysis

The continuous variables, such as length of hospital stay and hospitalization costs, were not normally distributed and were therefore analyzed using the non-parametric rank-sum test. Count data processing: Count data (such as the incidence of malnutrition risk in different age groups, the number of cases with high malnutrition risk in different diseases, and the proportion of nutritional support methods) are represented as “number (percentage).” The analysis of differences between groups was conducted using the chi-square test (*χ*^2^ test). Specifically, this includes analyzing the differences in the incidence of malnutrition risk among children in different age groups (0–3 years old, 3–6 years old, 6–13 years old, 13–18 years old); comparing the incidence of high malnutrition risk in different disease groups (hepatobiliary diseases, inflammatory bowel diseases, chronic gastroenteritis, etc.); and analyzing the differences in the proportion of nutritional support methods (enteral nutrition, parenteral nutrition) between the high-risk group and the non-high-risk group, as well as the differences in the rehospitalization rate between the two groups of children. Statistical analysis was performed using SPSS 25.0 (IBM, Chicago, IL, USA). A two-sided *P*-value of <0.05 was considered statistically significant.

### Malnutrition risk screening tool

The malnutrition risk assessment was performed using the above-mentioned MPMRST within 24 h of admission. Signed consent was obtained from the children's parents. The total score ranges from 0 to 9 points, including three parts: disease risk, dietary intake, and growth and development status. The details were as follows: (1) underlying disease (no, possible, and existent, with a score of 0, 2 and 3 points, respectively); (2) dietary intake (within 1 week; good, ≥50% reduction, and no intake, with a score 0, 2, and 3 points, respectively); (3) growth and development (the anthropometric parameters were classified based on World Health Organization (WHO) standards in 2007, *z*-score for weight for age (WAZ) for children aged <5 years, and *z*-score for body mass index for age (BAZ) for children aged 5–19 years. In addition, −2 ≤ *z* ≤ 2 was scored as 0 point, 2 < *z* < 3 or −3 < *z* < −2 as 1 point, and *z* > 3 or *z* < −3 as 3 points. Finally, the sum of the three scores was the total score, of which ≥2 was considered to be at risk of malnutrition and <2 was considered as no risk of malnutrition. At the same time, a score of ≥4 was considered as high malnutrition risk, and a score of <4 was considered as non-high malnutrition risk (Table 1).

**Table 1:** The scoring content of the modified pediatric malnutrition risk screening scale.

Item	Content	Score
1. Dietary intake	No change in diet and good intake	0
	≥50% reduction in recent dietary intake	2
	No nutrient intake	3
2. Impact of disease	No	0
	Possible (minor surgery, gastroesophageal reflux, eating behavior problems, celiac disease, heart disease, psychiatric disorders, single food allergy, cleft lip and palate, respiratory syncytial virus infection/intolerance)	2
	Existent (dysphagia, major surgery, congenital metabolic diseases, hepatobiliary disease, intractable diarrhea, Crohn's disease, burns, renal failure, intestinal failure, severe trauma, multiple food allergies, cystic fibrosis)	3
3. Growth and development status	−2 ≤ *z* ≤ 2	0
	2 < *z* < 3 or −3 < *z* < −2	1
	*z* > 3 or *z* < −3	3

Total score = (Score of dietary intake + Score of impact of disease + Score of growth and development status). The sum of the three scores was the total score, of which ≥2 was considered to be at risk of malnutrition and <2 was considered as no risk of malnutrition. At the same time, a score of ≥4 was considered as high malnutrition risk, and a score of <4 was considered as non-high malnutrition risk.

## Results

### Malnutrition risk screening rate

A total of 1,218 children were hospitalized in the gastroenterology department, and 18 patients were lost due to transfer to other wards or the Pediatric Intensive Care Unit (PICU) within 24 h. Among the 18 excluded cases, 55.6% (10/18) had high nutritional risk. However, after inclusion, the overall high-risk rate only increased from 13.67% to 14.29%, which did not affect the research conclusion. Finally, a total of 1,200 cases were included in this study (726 males and 474 females, aged from 1 month and 5 days to 17 years and 6 months). According to the MPMRST, 638 patients (53.17%) were identified as at risk of malnutrition, and 562 patients (46.83%) were not at risk of malnutrition. Of the 1,200 patients, 164 patients (13.67%) were classified as high risk, and 1,036 patients (86.33%) were classified as non-high risk (Tables 2, 3).

**Table 2:** The risk of malnutrition in 1,200 hospitalized children.

Group	*N* (cases)	Proportion (%)
At risk of malnutrition (≥2 points)	638	53.17
No risk of malnutrition (<2 points)	562	46.83
Total	1,200	100

**Table 3:** The risk degree of malnutrition in 1,200 hospitalized children.

Group	*N* (cases)	Proportion (%)
High risk of malnutrition (≥4 points)	164	13.67
Non-high risk of malnutrition (<4 points)	1,036	86.33
Total	1,200	100

### Malnutrition risk in different ages

In this study, all children were divided into four groups: 0–3 years old (infancy), 3–6 years old (preschool-age), 6–13 years old (school-age), and 13–18 years old (adolescence). The risk of malnutrition was significantly different between age groups (*χ*^2^ = 59.91, *P* < 0.05), with the highest in infancy followed by the preschool-age group (Table 4).

**Table 4:** Malnutrition risk in hospitalized children of different ages.

Age	Number of cases (*n*)	At risk of malnutrition [*n* (%)]	No risk of malnutrition [*n* (%)]	*χ* ^2^	*P*
0–3 years	445	257 (57.6)	188 (42.4)	59.91	<0.05
3–6 years	233	126 (54.1)	107 (45.9)		
6–13 years	489	240 (49.1)	249 (50.9)		
13–18 years	33	15 (45.5)	18 (54.5)		
Total	1,200	638 (53.2)	562 (46.8)		

### Malnutrition risk in hospitalized children with different diseases

The prevalence of high malnutrition risk significantly differed among hospitalized children with different diseases (*P* < 0.001). Specifically, the rates were as follows: 67.9% in anorexia nervosa, 35.7% in inflammatory bowel disease, 18.2% in esophageal stricture, 11.7% in chronic gastritis, 8.1% in hepatobiliary diseases, and 5.9% in other conditions (Table 5).

**Table 5:** Malnutrition risk in hospitalized children with different diseases.

Disease	Number of cases (*n*)	High risk (*n*)	Non-high risk (*n*)	Malnutrition risk (%)	*χ* ^2^	*P*
Hepatobiliary disease	122	10	112	8.1	117.45	<0.001
Inflammatory bowel disease	14	5	9	35.7		
Chronic gastroenteritis	861	101	760	11.7		
Anorexia nervosa	56	38	18	67.9		
Esophageal stenosis	11	2	9	18.2		
Others	136	8	128	5.9		

### Nutritional support for hospitalized children with different risk levels

Nutritional supports were not associated with the risk level of malnutrition (*P* = 0.056). However, parental nutrition support was significantly associated with a high level of risk (*P* = 0.027). The comparison of nutritional support for hospitalized children with different risk levels of malnutrition is presented in Tables 6 and 7.

**Table 6:** Comparison of nutritional support for hospitalized children with different risk levels of malnutrition.

Group	No nutritional support (*n*)	Nutritional support (*n*)	*χ* ^2^	*P*
Non-high risk (*n* = 1,036)	810	226	6.014	<0.0
High risk (*n* = 164)	114	50		
Total (*n* = 1,200)	924	276		

**Table 7:** Comparison of nutritional support ways for hospitalized children with different risk levels of malnutrition.

Group	Enteral (*n*)	Parental (*n*)	*χ* ^2^	*P*
Non-high risk (*n* = 226)	204	22	14.484	<0.05
High risk (*n* = 50)	35	15		
Total	239	37		

### Nutritional support for hospitalized children with different risk levels of malnutrition

The antibiotics usage frequency was not related to risk levels of malnutrition (*P* = 0.942). High risk level of malnutrition was associated with a significantly longer hospital stay, a higher hospital cost, and a higher rehospitalization rate (all *P* < 0.005) (Table 8).

**Table 8:** Comparison of outcomes of hospitalized children with different risk levels of malnutrition.

Outcomes	Non-high risk	High risk	*z*/*t*	*P*
Duration of hospital stay (day, $\bar{x}\pm s$)	3.77 ± 0.69	4.58 ± 0.29	−16.702^a^	0.000
Antibiotic usage frequency (*n*/%)	116/11.06	17/11.26	0.005^b^	0.942
Hospital cost (thousand yuan, $\bar{x}\pm s$)	5.24 ± 0.61	6.92 ± 0.27	−2.81^a^	0.004
Rehospitalization rate (*n*/%)	71/6.77	21/13.91	9.503^b^	0.002

^a^ Results of the Mann–Whitney–Wilcoxon test.

^b^ Results of the chi-square test.

## Discussion

In this study, the total malnutrition risk in children hospitalized was 53.17%. It was similar to the findings from a previously published study on 2,632 hospitalized children (15). However, the high risk level of malnutrition, i.e., 13.67%, was lower in this study than that in the previous studies (17–19). We speculated that the difference was partially due to the population with digestive system diseases in the present study, which was different from the previous study focused on the children with diseases of other systems (15). This may also be partially due to parents’ greater awareness of health and higher attention to the nutritional status of children in this study from a region with a higher economic level, which contributes to the prevention of malnutrition (20).

In this study, younger age was associated with a higher risk of malnutrition. The infants aged 0–3 years were at the highest risk, followed by the preschool-age group aged 3–6 years of age. This was similar to the findings of malnutrition risk in children aged 0–1 year, which was higher than that in other age groups (15). The result of this study indicated that the potential risk of malnutrition should be highly vigilant in infancy due to the immature digestive system and autoimmune function, as well as rapid growth and development, leading to the susceptibility to gastrointestinal dysfunction and/or infectious diseases. Therefore, careful screening and assessment of malnutrition risk for children at different ages are important.

In contrast to the result in this study, a published study on 4,728 hospitalized children in Suzhou, China, showed that the risk of malnutrition was the highest in children over 7 years old, whereas it was the lowest in children aged 2–3 years old (21). Possible explanations for this contradiction might be the use of a different screening tool in this study and the focus on patients hospitalized specifically for digestive diseases, as opposed to all hospitalized children with internal and surgical diseases. The contradictory results indicated that the association of age and nutritional risk needs to be investigated further.

We further analyzed the risk level of malnutrition in children with different disorders in this study. Anorexia nervosa was associated with a higher risk level of malnutrition, followed by inflammatory bowel disease and esophageal stenosis. Interestingly, hepatobiliary disease and chronic gastroenteritis were at a lower risk level of malnutrition. The result demonstrated that subjective restriction of food intake for weight loss implies tremendous risk of severe malnutrition and secondary endocrine and metabolic changes; timely assessment of nutritional risk and continuous dynamic monitoring is important for the prognosis in children with anorexia nervosa. In this study, the risk of malnutrition was much lower in children with inflammatory bowel disease than that in adults reported by Lin Maowei (21) and Zhang Qian (22). This finding needs to be further investigated in future studies with a larger sample.

Similar to the result of a random sample survey, 25% of hospitalized children with digestive system diseases were intervened with either enteral or parenteral nutrition supports (23, 24). Moreover, more children with a high risk level need nutritional support compared with the non-high risk group. In this study, once a child was identified with high nutritional risk, a nutritional support team (NST) was involved to provide multidisciplinary consultation. For high-risk children, nutritional assessment is conducted, and a nutrition support plan is formulated based on the assessment results. This mainly includes enteral (oral or tube feeding of formula milk)/parenteral (intravenous infusion through peripheral veins or central veins of hypertonic glucose, fat emulsions, compound amino acid solutions, multiple vitamins and trace element solutions, etc.) or combined enteral and parenteral nutrition support. In addition, parenteral nutrition support was used more frequently in children at high risk of malnutrition than in those who were non-high risk in this study. This suggests that the application of the MPMST to assess the nutritional risk in children may be helpful for a reasonable method of nutritional support and contribute to preventing further nutritional disorders.

Nutritional risk status is an important predicting factor of clinical outcomes (25). In this study, we further compared the outcomes between groups with different levels of malnutrition risk. To compare easily, we divided all the children into two groups: high risk with a total score of ≥4 and non-high risk with a total score of <4. The result showed that a high risk level of malnutrition was associated with a significantly longer duration of hospital stay, a higher hospital cost, and a higher rehospitalization rate, which were consistent with the findings of previous studies (13, 26). Therefore, more concerns, prompt nutritional interventions, and dynamic monitoring of nutritional status are crucial for children with a high risk level of malnutrition in the gastroenterology department (27).

This study has limitations. It was a single-center study and did not include comparisons with other nutritional risk screening tools. In addition, the children were not followed over time, and the assessment of the tool on predicting outcomes was not applied.

## Conclusion

The malnutrition risk was prevalent in hospitalized children with digestive system diseases. Children with anorexia nervosa and inflammatory bowel disease were at a higher risk level of malnutrition. A high risk level of malnutrition was associated with a significantly longer duration of hospital stay, a higher hospital cost, and a higher rehospitalization rate. Therefore, prompt and appropriate malnutrition risk screening and assessment may provide evidence for clinical nutrition intervention, and reasonable nutritional supports may improve prognosis.

## Data Availability

The original contributions presented in the study are included in the article/Supplementary Material; further inquiries can be directed to the corresponding author.
